# The Research Progress in Immunotherapy of Tuberculosis

**DOI:** 10.3389/fcimb.2021.763591

**Published:** 2021-11-15

**Authors:** Jie Mi, Yan Liang, Jianqin Liang, Wenping Gong, Shuyong Wang, Junxian Zhang, Zhiming Li, Xueqiong Wu

**Affiliations:** Tuberculosis Prevention and Control Key Laboratory/Beijing Key Laboratory of New Techniques of Tuberculosis Diagnosis and Treatment, Senior Department of Tuberculosis, The 8th Medical Center of PLA General Hospital, Beijing, China

**Keywords:** immunotherapy, tuberculosis, immunoactive substances, tuberculosis therapeutic vaccines, chemical agents, cellular therapy

## Abstract

Tuberculosis (TB) is a serious public health problem worldwide. The combination of various anti-TB drugs is mainly used to treat TB in clinical practice. Despite the availability of effective antibiotics, effective treatment regimens still require long-term use of multiple drugs, leading to toxicity, low patient compliance, and the development of drug resistance. It has been confirmed that immune recognition, immune response, and immune regulation of *Mycobacterium tuberculosis* (*Mtb*) determine the occurrence, development, and outcome of diseases after *Mtb* infection. The research and development of TB-specific immunotherapy agents can effectively regulate the anti-TB immune response and provide a new approach toward the combined treatment of TB, thereby preventing and intervening in populations at high risk of TB infection. These immunotherapy agents will promote satisfactory progress in anti-TB treatment, achieving the goal of “ultra-short course chemotherapy.” This review highlights the research progress in immunotherapy of TB, including immunoreactive substances, tuberculosis therapeutic vaccines, chemical agents, and cellular therapy.

## 1 Introduction

Tuberculosis (TB) is a leading infectious disease caused by *Mycobacterium tuberculosis* (*Mtb*), which invades the host. The World Health Organization (WHO) estimated 9.9 million new cases and 1.28 million deaths in 2020 (WHO, 2021). Despite the availability of antibiotics and effective treatment regimens, there are still many inevitable problems during chemotherapy, such as the long course of treatment, severe adverse reactions, poor compliance, and multidrug resistance. Therefore, anti-TB treatments are facing significant challenges worldwide.

It is well known that immune recognition, immune response, and immune regulation to *Mtb* determine the occurrence, development, and outcome of the disease. The immune response of the body to TB results from the interactions of diverse innate and adaptive immune cells and is determined by genetic and environmental factors of the bacteria and host. At the initial stage of infection, robust innate immunity plays a pivotal role in the early clearance of *Mtb*. Macrophages, natural killer cells, dendritic cells, γδ T cells, neutrophils, and other phagocytes together build the first-line of defense, in which macrophages are the most essential cells in resisting *Mtb* (Sia et al., 2015). *Mtb* mainly survives and proliferates in alveolar macrophages and other innate immune cells of the host. The interaction between *Mtb* and immunity is a dynamic game process, leading to different outcomes: (1) When the invasiveness of *Mtb* is weaker than the host immunity, the alveolar macrophages directly kill and eliminate *Mtb* (Korbel et al., 2008; Cadena et al., 2016). After that, macrophages, NK cells and other innate immune cell populations can produce “trained immunity,” and the immune system can mount a faster and more effective protective immune response after the second invasiveness of *Mtb* (Divangahi et al., 2021); (2) If the invasiveness of *Mtb* is balanced with host immunity, *Mtb* may turn into a dormant state, presenting immune escape and a symbiotic state with the host (Gong and Wu, 2021); (3) When the invasiveness of *Mtb* is stronger than the host immunity, *Mtb* will replicate in the granuloma, which may undergo caseous necrosis, liquefaction, and cavitation, leading to a spread of *Mtb* and an initiation of active TB (Khan et al., 2016; de Martino et al., 2019). Thus, adaptive immune responses play an essential role in anti-TB immune defense. Since the protective responses of the host against TB are based on the production of innate immune cells and the interaction between activated macrophages and specific T cells, enhancing protective immunity or regulating adaptive immune responses against TB may be valuable adjuvant treatments for advanced disease. Therefore, reasonable anti-TB chemotherapy combined with immune modulators will help adjust the immune status of the patient and enhance the therapeutic effect of chemical drugs on TB. In general, anti-TB immunotherapy mainly includes activating immune activity, enhancing protective immunity, and suppressing adverse immune responses and inflammatory damage. The research and development of TB-specific immunotherapy agents can effectively regulate the anti-TB immune response, provide a new way for the combined treatment of TB, prevent and intervene in populations at high risk of TB infection, which will make TB treatment achieve a significant effect, and achieve the goal of “ultra-short course chemotherapy.”

This review highlights the research progress in the immunotherapy of TB including immunoactive substances, tuberculosis therapeutic vaccines, chemical agents, and cellular therapy (**Tables 1**, **2**). We believe that immunotherapy has a high potential to prevent the activation of latent *Mtb* and treat patients with active TB.

**Table 1:** Summary of the development of immunoactive substances, vaccines, chemical agents, and cellular therapies.

Type	Name		Immune Mechanism	Immunotherapeutic Effect	Model of Study	Clinical Trials			Reference
						Phase	CTR Number^a^	Status^b^	
Immunoactive substances	Cytokines	IL-2	Promote the proliferation and transformation of CD4^+^ T cells and NK cells	Improve sputum bacteria negative rate, but the improvement of radiographic changes was not significant	Human	II, III	NCT03069534	Unknown	(Johnson B. et al., 1998; Johnson B.J. et al., 1998; Johnson et al., 2003; Zhang et al., 2018)
						IV	NCT04766307	Recruiting	
		GM-CSF	Reduce the growth of *Mtb* in human mononuclear macrophages	Promote the sputum bacteria turned negative; reduce the pulmonary bacterial burden, improve the efficacy of isoniazid and rifampin	Human;	II	——	——	(Pedral-Sampaio et al., 2003; Zhang et al., 2012)
					Mouse				
		IL-24	Activate the IL-24 receptor signaling pathway of CD8^+^ T cells to produce IFN-γ	Improve survival rate, reduce bacterial counts, protect mice from *Mtb* infection	Mouse	——	——	——	(Ma et al., 2011)
		IL-32	Promote the up-regulated expression of TNF-α, IL-1β and IL-8. The anti-TB effect depends on the relative abundance of IL-32 isoforms	Reduce the *Mtb* in the lungs in a mouse model	Mouse	——	——	——	(Kim et al., 2005; Netea et al., 2008; Koeken et al., 2019)
	Small molecule active peptides	AMPs	The bactericidal effect on *Mtb* is independent of the type of strain	Reduce lung inflammation and bacterial load	Mouse	——	——	——	(Fehlbaum et al., 2000; Rivas-Santiago et al., 2013; Rivas-Santiago et al., 2015; Silva et al., 2016b; Sharma et al., 2018)
		Thymopentin	Increase the secretion of Th1 and Th17 cells, decrease Th2, Treg responses, and PD-1 expression	Promote sputum bacteria negative rate and lesion absorption	Human;	——	——	——	(Yuan et al., 2016; Bohua Wu and Chen, 2018)
					Mouse				
	Immune blocker	IL-4	Inhibit the activity of immune molecules that are harmful to the body, and bias the immune response toward Th1	Decrease the lung bacterial counts and improve the survival rate in a mouse model with TB	Human;	II	NCT01638520	Unknown	(Hernández‐Pando et al., 2006; Buccheri et al., 2007; Beamer et al., 2008; Singh et al., 2013; Segueni et al., 2016; Gupta et al., 2017)
					Mouse				
Therapeutic vaccines	Inactivated TB vaccines	*M.* vaccae	Enhance the cellular immune function of patients with anti-TB infection	Improve the sputum bacteria negative rate effectively, but the effects on the absorption of lesions, cavity closure, and mortality are not significant	Human	III	NCT01979900	Completed	(Efremenko et al., 2013; Huang and Hsieh, 2017)
							NCT01977768	Completed	
		MIP vaccine	Induce clock like receptor signal pathways to activate innate immunity and stimulate T cell immune response	Improve the sputum negative rate, have the adverse reaction of Kaposi sarcoma	Human	III	NCT00341328	Completed	(Mayosi et al., 2014; Sharma et al., 2017)
							NCT00265226	Completed	
		DAR-901 (Mk)	Enhance Th1 cytokine response, improve immunity	Improve sputum bacteria negative rate, promote lesion absorption	Human	II	NCT02712424	Completed	(Gröschel et al., 2014; Munseri et al., 2020)
		RUTI	Induce mixed Th1/Th2/	Direct use of RUTI leads to immune damage; induce an immune response in LTBI volunteers	Human; Mouse	II	NCT02711735	Recruiting	(Cardona et al., 2005; Nell et al., 2014)
			Th3, polyantigenic response with no local or systemic toxicity						
							NCT04919239	Not yet recruiting	
	TB subunit vaccines	BCG-PSN	Enhance cellular immunity and humoral immunity	Mainly used in non-TB immunocompromised diseases	Human	——	——	——	(Nasr et al., 2018; Yan et al., 2019)
		*Mtb*72f/AS01E	Elicit high magnitude M72-specific humoral and CD4^+^ T cell responses	Provide 54% protection for LTBI adults	Human	II	NCT01755598	Completed	(Leroux-Roels et al., 2013; Van Der Meeren et al., 2018)
							NCT00397943	Completed	
						I	NCT00730795	Completed	
		H56: IC31	Induce IgG and CD4^+^ T cell expression of Th1 type cytokines; low dose vaccination can promote the secretion of TNF-α+IL-2+H56-specific memory CD4^+^ T cells	Prevent bacterial reactivation, reduce bacterial load	Human	II	NCT03512249	Recruiting	(Luabeya et al., 2015)
						I	NCT02503839	Unknown	
		ID93/GLA-SE	Induce a strong and durable Th1 type immune response	Prolong survival time, reduce the bacterial burden and pathological damage, enhance chemotherapy effect	Human;	II	NCT03806686	Unknown	(Bertholet et al., 2010; Baldwin et al., 2021)
					Mouse;				
					Cynomolgus monkey				
						I	NCT03806699	Unknown	
		AEC/BC02	Induce long-term antigen-specific cellular immune responses	Have a significant inhibitory effect on latent *Mtb*, reduce the degree of lesions	Human	I	NCT04239313	Active, not recruiting	(Lu et al., 2015)
	DNA vaccines	GX-70	The only TB DNA vaccine to enter clinical trials	The clinical trial of tolerability, safety, and immunogenicity in PTB patients is ongoing	Human	I	NCT03159975	Withdrawn	(Gong et al., 2018)
		Ag85a/b	Induce a strong Th1 cellular immune response and moderate levels of antibodies	Reduce lung tissue lesions and organ colony with no adverse reactions	Mouse	——	——	——	(Liang et al., 2012)
Chemical agents	Vitamin D		Inhibit *Mtb* growth and the secretion of pro-inflammatory cytokines; induce LL-37 or autophagy-related proteins Beclin-1 and Atg5	Low sputum negative conversion rate	Human	Not Applicable	NCT01992263	Not yet recruiting	(Wallis and Zumla, 2016; Mourik et al., 2017; Bekele et al., 2018)
						IV	NCT02169570	Unknown	
							NCT02464683		
						III	NCT02880982	Active, not recruiting	
						II	NCT02968927	Unknown	
	Quercetin and polyvinylpyrrolidone		Promote the growth of endothelial cells, induce the activation of microcirculation in inflammation sites, and	Reduce inflammation and blood coagulation, reduce lung bacterial counts and cavities	Human;	——	——	——	(Butov et al., 2016)
					Mouse				
	Bergenin		Activate MAPK, ERK1/2, and SAPK/JNK pathways, induce CD4^+^ and CD8^+^ T cells to produce IFN-γ, TNF-α, IL-12 and IL-17	Reduce the pathological damage and bacterial burden, shorten the treatment time	Mouse	**——**	——	——	(Kumar et al., 2019)
	Allicin		Activate the MAPK and SAPK/JNK pathways to produce IL-1β and IL-12; induce Th1 responses and inhibit the phosphorylation of p38-MAPK	Reduce the bacterial load in the lungs of mice with TB	Mouse	——	——	——	(Oosthuizen et al., 2017; Dwivedi et al., 2019)
	Ursolic acid		Produce NO and reactive oxygen species, inhibit TGF-β expression, induce TNF-α expression, and activate macrophages	Direct inhibit the mycobacteria, reduce the bacterial load in mouse models with sensitive *Mtb* and MDR-*Mtb*	Mouse	——	——	——	(López-García et al., 2015)
	Oleanolic acid								
	Chicoric acid		The combination therapy can increase the NO production, CD14, and HLA-DR expression	Inhibit the growth of *Mtb*	Mouse	——	——	——	(Abd-Nikfarjam et al., 2018)
	Retinoic acid								
	Curcumin		Enhance *Mtb* clearance by differentiated THP-1 human monocytes and primary human alveolar macrophages	The anti-TB effect needs to be further clarified	Mouse	——	——	——	(Bai et al., 2016)
	Loperamide		Up-regulate the expression of BPI and antimicrobial peptide LL37 genes, block the intracellular calcium influx, activate μ-opioid receptors	Reduce the bacterial load, avoid inflammatory damage	Mouse	——	——	——	(Juárez et al., 2018)
	Phosphatidylinositol mannosides		Bind to macrophages, regulate the production of cytokines and reactive radical species, stimulate the early endoplasmic fusion, activate NKT cells produce IFN-γ	Have adjuvant activity, promote the granulomatous formation, enhance the immune response	Mouse	——	——	——	(Patil et al., 2015)
Cellular therapy	Mesenchymal stem cells		Have advantages of anti-inflammatory, immune regulation, promoting tissue regeneration and repair, and low immunogenicity, but may activate dormant *Mtb* and assist in immune escape	The sputum of patients turned negative, the lung cavity narrowed or closed, and the cure rate significantly increased, but may cause extrapulmonary TB	Human	II	NCT04493918	Recruiting	(Erokhin et al., 2008; Skrahina et al., 2012; Skrahin et al., 2014; Hoagland et al., 2016; Khan A. et al., 2016)
						I	DRKS00000763	Recruiting	
	γδ T cells		Lyse cells, produce cytokines and regulate immune cells	Inhibit or kill intracellular *Mtb*, reduce the bacterial load on the lungs and extrapulmonary organ to reduce lung tissue damage	Human;	I	NCT03575299	Recruiting	(Chen et al., 2013)
					Rhesus macaques				
	Cytokine-induced killer cells		Are nonrestrictive killer cells	The sputum culture and smear of patients turned negative without liver damage	Human	——	——	——	(Xu et al., 2015)
	Invariant NKT cells		Release IFN-γ, activate macrophages to secrete TNF-α and NO, enhance the killing effect of phagosomes and lysosomes on *Mtb*	Limit *Mtb* proliferation *in vivo*	Human	I, II	NCT03551795	Unknown	(Sada-Ovalle et al., 2008; Rothchild et al., 2014)

a CTR number, Clinical trial registration number; NCT number, ClinicalTrials.gov Identifier; DRK number, German Clinical Trials Registry; Only the latest clinical trials were listed here.

b The status of each trial was offered by ClinicalTrials.gov data bank (https://clinicaltrials.gov/ct2/home), German Clinical Trials Registry (https://www.drks.de/drks_web/navigate.do?navigationId=search&reset=true). The data were obtained on August 18, 2021.

**Table 2:** The development of clinical trials in immunoactive substances, vaccines, chemical agents, and cellular therapies.

Intervention	Condition^a^	Sponsors and collaborators^b^	Number Enrolled	Phase	CTR Number^c^	Status
IL-2	MDR-TB	FAHNMU	500	II, III	NCT03069534	Unknown
	PTB	BCH	1100	IV	NCT04766307	Recruiting
IL-4	PTB	NUH, NU	32	II	NCT01638520	Unknown
*M.* vaccae	TB	AHZLBP, GXCDCP, LZCDCP, RSCDCP, LCCDCP, JCJCDCP, NIFDC, AFMMU, SRPICC	10000	III	NCT01979900	Completed
	TB	Immunitor LLC, NMU, Immunitor USA Inc., UOS, LRTD	152	III	NCT01977768	Completed
MIP vaccine	TB	MST	300	III	NCT00341328	Completed
	TB	MST	1020	III	NCT00265226	Completed
DAR-901 (Mk)	TB	DHMC, MUHAS	625	II	NCT02712424	Completed
RUTI	MDR-TB	Archivel Farma S.L., LSHTM	27	II	NCT02711735	Recruiting
	PTB	Archivel Farma S.L.	140	II	NCT04919239	Not yet recruiting
*Mtb*72f/AS01E	TB	GSK, Aeras	3575	II	NCT01755598	Completed
	TB	GSK	110	II	NCT00397943	Completed
	TB	GSK, CC	12	I	NCT00730795	Completed
H56: IC31	PTB	Aeras, SSI, SATVI, UCTL, TASKAS, AINPC, NIMR, OSR, EDCTP	900	II	NCT03512249	Recruiting
	TB	AMDR, UO, SSI, HUH	39	I	NCT02503839	Unknown
ID93/GLA-SE	TB	Quratis Inc., IDRI	107	II	NCT03806686	Unknown
	TB	Quratis Inc.	36	I	NCT03806699	Unknown
AEC/BC02	TB	AHZLBP	30	I	NCT04239313	Active, not recruiting
GX-70	PTB	YU	0	I	NCT03159975	Withdrawn
Vitamin D	TB	CU, AMC	200	Not Applicable	NCT01992263	Not yet recruiting
	PTB	DUHS	435	IV	NCT02169570	Unknown
	PTB	INER	60	IV	NCT02464683	Unknown
	LTBI	QMUL, UCT	1743	III	NCT02880982	Active, not recruiting
	TB	AINPC	200	II	NCT02968927	Unknown
Mesenchymal stem cells	STB	AJR, IU	20	II	NCT04493918	Recruiting
	MDR-TB, XDR-TB	RRPCPT	40	I	DRKS00000763	Recruiting
γδ T cells	MDR-TB	YZN, SZTPH	45	I	NCT03575299	Recruiting
Invariant NKT cells	TB with malignant solid tumor	ZXY, SHPHCC	8	I, II	NCT03551795	Unknown

a TB: Tuberculosis; PTB: Pulmonary Tuberculosis; LTBI: Latent Tuberculosis Infection; STB: Spinal Tuberculosis; MDR-TB: Multidrug Resistant Tuberculosis; XDR-TB: Extreme drug Resistant (XDR) Tuberculosis.

b AFMMU, Air Force Military Medical University (China); AHZLBP, Anhui Zhifei Longcom Biologic Pharmacy Co., Ltd. (China); AINPC, The Aurum Institute NPC (South Africa); AJR, Ahmad Jabir Rahyussalim (Indonesia); AMC, Arogyavaram Medical Centre (USA); AMDR, Anne Margarita Dyrhol Riise (Norway); BCH, Beijing Chest Hospital (China); CC, Corixa Corporation (USA); CU, Cornell University (USA); DHMC, Dartmouth-Hitchcock Medical Center (USA); DUHS, Dow University of Health Sciences (Pakistan); FAHNMU, The First Affiliated Hospital with Nanjing Medical University (China); GSK, Glaxo-SmithKline Biologicals (UK); GXCDCP, Guangxi Center for Disease Control and Prevention (China); HUH, Haukeland University Hospital (Norway); INER, Instituto Nacional de Enfermedades Respiratorias (Mexico); IU, Indonesia University (Indonesia); JCJCDCP, Jin Chengjiang Center for Disease Control and Prevention (China); LRTD, Lisichansk Regional Tuberculosis Dispensary (Ukraine); LSHTM, London School of Hygiene and Tropical Medicine (UK); LCCDCP, Liucheng County Disease Control and Prevention (China); LZCDCP, Liuzhou Center for Disease Control and Prevention (China); MST, Ministry of Science and Technology (India); MUHAS, Muhimbili University of Health and Allied Sciences (Tanzania); NIFDC, National Institutes for Food and Drug Control (China); NMU, National Medical University (Ukraine); NU, National University (Singapore); NUH, National University Hospital (Singapore); QMUL, Queen Mary University of London (UK); RRPCPT, Republican Research and Practical Center for Pulmonology and Tuberculosis (Minsk Belarus); RSCDCP, Rongshui County Disease Control and Prevention (China); SATVI, South African Tuberculosis Vaccine Initiative (South Africa); SHPHCC, Shanghai Public Health Clinical Center (China); SRPICC, Simoon Record Pharma Information Consulting Co., Ltd. (China); SSI, Statens Serum Institut (Denmark); SZTPH, Shenzhen Third People’s Hospital (China); UCT, University of Cape Town (South Africa); UCTLI, University of Cape Town Lung Institute (South Africa); UO, University of Oslo (Norway), UOS, University of Stellenbosch (South Africa); YU, Yonsei University (Korea); YZN, Zhinan Yin, Ph.D. (China); ZXY, Xiaoyan Zhang (China).

c CTR number, Clinical trial registration number; NCT number, ClinicalTrials.gov Identifier; DRK number, German Clinical Trials Registry; Only the latest clinical trials are listed here. TASKAS, TASK Applied Science (South Africa); AINPC, The Aurum Institute NPC (South Africa); NIMR, National Institute for Medical Research (Tanzania); OSR, Ospedale San Raffaele (Italy); EDCTP, European and Developing Countries Clinical Trials Partnership.

## 2 Immunoactive Substances

Immunoactive substances, produced by immune cells or other cells to exert an immune effect, mainly include cytokines (such as interferon, interleukin, and tumor necrosis factor), antibodies, lysozyme, and complement. In recent years, a growing number of studies have focused on the application of these immunoactive substances in the immunotherapy of TB.

### 2.1 Cytokines

Cytokines are proteins that coordinate innate and adaptive immune responses by affecting cell development, transport, and function. Currently, the cytokines used in clinical applications or clinical trials are recombinant human interferon (rhuIFN-γ), recombinant human interleukin-2 (rhuIL-2), α-tumor necrosis factor (TNF-*α*), and recombinant human granulocyte-macrophage colony-stimulating factor (rhuGM-CSF); however, cytokine immunotherapy has the shortcomings of short half-life and high cost.

#### 2.1.1 Interleukin-2

IL-2, a cytokine belonging to the Th1-type immune response, functions in immune activation and regulation. Studies have shown that IL-2 can induce differential expression of genes in peripheral blood mononuclear cells (PBMCs) stimulated with *Mtb*, and reduce or clear sputum bacteria in about 60% of patients with MDR-TB combined with chemotherapy (Johnson B. et al., 1998; Johnson B.J. et al., 1998). However, data from a double-blind, placebo-controlled clinical trial showed that daily intradermal injection of rhuIL-2 could not enhance bacillary clearance or improve symptoms in patients with drug-susceptible TB (Johnson et al., 2003). Therefore, the clinical results of rhuIL-2 combined with chemotherapy for refractory pulmonary TB (PTB) or MDR-TB are inconsistent. In addition, a meta-analysis showed that rhuIL-2 immunoadjuvant therapy was safe for patients with PTB/MDR-TB, and could promote the proliferation and transformation of CD4^+^ T cells and NK cells, improving the sputum bacterium-negative rate of patients with TB, however, there was no significant improvement in radiographic changes in patients with TB (Zhang et al., 2018). A multicenter, large-sample prospective clinical study of rhuIL-2 adjuvant therapy for MDR-TB for 24 months is ongoing in China (ClinicalTrials.gov Identifier: NCT03069534).

#### 2.1.2 Granulocyte-Macrophage Colony-Stimulating Factor

GM-CSF, a cytokine with immune activation and regulatory effects, is a monomer glycoprotein secreted by macrophages, T cells, mast cells, natural killer cells, endothelial cells, and fibroblasts. GM-CSF has been shown to reduce the growth of *Mtb* in human mononuclear macrophages (Denis et al., 1990). The results of a phase II clinical trial of rhuGM-CSF combined with anti-TB chemotherapy in the treatment of active PTB (APTB) showed that rhuGM-CSF adjuvant immunotherapy had better safety and tolerance in patients, and the sputum bacteria rapidly turned negative in the eighth week of treatment (Pedral-Sampaio et al., 2003). In addition, immunotherapy with IL-2 and GM-CSF in the MDR-TB mouse model could increase the survival rate of mice and decrease the bacterial loads in the lung, spleen and lung lesions, which will improve the efficacy of first-line anti-TB drugs (isoniazid and rifampin) (Zhang et al., 2012). As a new gene therapy, recombinant GM-CSF adenoviruses (AdGM-CSF) in a mouse model could also significantly reduce pulmonary bacterial burden compared to conventional chemotherapy when administered in a single dose (Francisco-Cruz et al., 2016).

#### 2.1.3 Interleukin-24

IL-24, a novel tumor suppressor, has been designated as a member of the IL-10 cytokine family because of its conserved structure, chromosomal location, and cytokine-like properties. *Mtb* infection has been demonstrated to inhibit the expression of IL-24 in human PBMCs and decrease the levels of IL-24 in the sera of patients with TB, which may increase the susceptibility of TB and promote the development of chronic TB (Ma et al., 2011). IL-24 can activate the IL-24 receptor signaling pathway of CD8^+^ T cells to produce large amounts of interferon-γ (IFN-γ) to combat *Mtb*, which depends on the early involvement of neutrophils (Ma et al., 2011). The application of IL-24 in the treatment of the mouse TB model showed an anti-TB effect, suggesting that IL-24 might be a new potential immunotherapy (Ma et al., 2011).

#### 2.1.4 Interleukin-32

IL-32, a cytokine produced mainly by T cells, NK cells, and epithelial cells, is an important secretory protein involved in innate and adaptive immune responses that induce the production of critical inflammatory factors (such as TNF-α, IL-1β, IL-6, MIP-2, and IL-8) in macrophages to eliminate *Mtb* (Kim et al., 2005; Netea et al., 2008). Therefore, the amplification effect of IL-32 in innate immunity against TB can upregulate TNF-α and promote cell apoptosis. Recent studies have found that stimulation of human PBMCs with heat-killed *Mtb* could induce the production of a large amount of IL-32 to enhance the *Mtb* clearance ability of human monocyte-macrophages (Li et al., 2018). The amount of lung *Mtb* can be significantly reduced by human IL-32γ expressed by type II alveolar epithelial cells from transgenic mice (Bai et al., 2015). In addition, after the downregulation of endogenous IL-32 expression in human THP-1 macrophages by siRNA interference, intracellular inflammatory factors, such as TNF-α, IL-1β, and significantly, and the intracellular *Mtb* increased (Bai et al., 2010). In the *Mtb*-infected PBMCs of the healthy control group, IL-32γ was downregulated while IL-32β was upregulated, suggesting that IL-32 contributes to preventing *Mtb* infection, and this effect may depend on the relative abundance of IL-32 isoforms (Koeken et al., 2019). Thus, IL-32 is a promising new immunotherapy for establishing protective responses and inhibiting *Mtb* growth.

### 2.2 Anti-TB Antibodies

The role of humoral immunity in TB is controversial because anti-TB is generally believed to rely mainly on the cellular immune response. However, more studies have shown that antibodies also have a protective effect on anti-TB immunity in recent years. The application of *Mtb* antigen-specific antibodies could potentially induce an inflammatory response, phagosome maturation, and intracellular bactericidal activity, and can block the immune damage induced by harmful molecules (Teitelbaum et al., 1998; Hamasur et al., 2004b).

A meta-analysis showed increased susceptibility to TB in some antibody-deficient hosts (Rodríguez et al., 2005). The protective function of antibodies against different *Mtb* epitopes varies. For example, the lower the level of anti-LAM or AM antibodies in patients with TB, the faster the progress of TB and the higher the frequency of dissemination (Costello et al., 1992); human anti-HBHA IgM antibodies could prevent *Mtb* from entering the epithelial cells of patients with TB (Shin et al., 2006); anti-Ag85A IgG could reduce the risk of active TB, reduce cavities, and remove sputum bacteria (Liang et al., 2018).

In addition, passive injection of poly/monoclonal antibodies or serum against *Mtb* antigen could improve phagocytosis, regulate CD8^+^ T cells, and reduce tissue damage, lung inflammation, and bacterial load in mice. For example, *Mtb*-infected mice inoculated intratracheally or intranasally with anti-Acr IgA antibody or pretreated with hsIgA reduced lung colony counts and improved granulomatous formation (Williams et al., 2004; Alvarez et al., 2013); *Mycobacterium* surface protein heparin-binding hemagglutinin adhesive (HBHA) promoted *Mtb* dissemination in mice, while the passive transfer of anti-HBHA IgG3 McAb 4057D2 and IgG2a McAb 3921E4, or anti-LAM IgG could significantly reduce the *Mtb* extrapulmonary spread (Pethe et al., 2001; Hamasur et al., 2004a).

Immunoglobulin Y (IgY), a major antibody in the blood of poultry, reptiles, and lungfish, is a potential immunoglobulin for TB immunotherapy with functions similar to IgG (Sudjarwo et al., 2017b). Similar to other immunoglobulins, IgY is a class of recognition proteins formed by the immune system in response to foreign substances. Sudjarwo *et al*. found that IgY could significantly increase the proliferation of rat PBMCs and secretion of IL-2 and IFN-γ in PBMCs, suggesting that the pharmacological activity of IgY against *Mtb* may be mediated by regulating the production of cytokines (Sudjarwo et al., 2017a).

### 2.3 Small Molecule Active Peptides

Small molecule active peptides, consisting of 2 to 15 amino acids with a simple structure and small molecular weight, can directly enter the cells through the skin, blood-brain, placenta, and gastrointestinal barriers to exert their biological activity.

Antimicrobial peptides (AMPs) are small, cationic, and amphiphilic peptides produced by organisms. They have antimicrobial activity as well as chemotactic, autophagic, and immunoregulatory activities (Hancock and Lehrer, 1998; Yamasaki and Gallo, 2008). Previous studies have identified many AMPs that have a potential immunotherapeutic effect on *Mtb* infection, and their bactericidal effect on *Mtb* was independent of the type of *Mtb* strain (e.g., whether they are resistant to drugs or not) (AlMatar et al., 2018). The essential amino acid L-isoleucine and its analogs have been shown to induce *β*-defensins through the activation of the transcription factor NF-κB, and using L-isoleucine to treat the mouse model with PTB could increase the expression of mβd-3 and -4 genes, as well as reduce the bacterial load and pulmonary inflammation (Fehlbaum et al., 2000); nano-encapsulated synthetic Magainin-I analog peptide could reduce the amount of viable *Mtb* by up to approximately 3.03-log CFU and enhance host defense mechanism by averting bacteria-induced inhibition of phagosomal-lysosome fusion and apoptosis (Sharma et al., 2018); and recombinant human neutrophil peptide-1 (HNP-1), human beta defensin-2 (HBD-2), HNP-1/HBD-2, LL-37 derived peptide HHC-10 or LLKKK18, and natural defense regulatory factors (IDR HH2 and IDR-1018) were used to treat the murine model of TB, resulting in diminished bacillary load and pulmonary inflammation (Rivas-Santiago et al., 2013; Rivas-Santiago et al., 2015; Silva et al., 2016b). In addition, several other AMPs have also shown anti-TB activity, including amphiphilic α-helical peptide D-V13 K, D-LAK analogs, bacteriocins (Bcn1–Bcn5), nisin A, lactoferrin, HBD variants, PR-39, and 1-C134mer (Silva et al., 2016a).

Thymopentin is a synthetic pentapeptide with immunological activity. In a BALB/c mouse model treated with thymopentin, Th1 and Th17 cells in the peripheral blood increased significantly, while Th2, Treg responses, and programmed cell death protein-1 (PD-1) expression decreased, with no significant side effects when compared with the control group (Yuan et al., 2016). In addition, patients with TB treated with thymopentin combined with chemotherapy showed a synergistic effect with symptom improvement, promoting sputum-negative conversion and lesion absorption (Wu et al., 2018).

### 2.4 Immune Blocker

Long-term chronic inflammation caused by active pulmonary TB can lead to Th1 and Th2 immune imbalance, immunosuppression, or T cell depletion. The application of immune blockers to block the action of harmful immune molecules can achieve immunotherapeutic purposes. For example, a mouse TB model treated with an anti-IL-4 antibody could block the secretion of the Th2 cytokine IL-4, shift the immune balance toward a protective Th1 response, and reduce the bacillary load in mouse spleens and lungs (Buccheri et al., 2007). In June 2012, a randomized, double-blind, placebo-controlled phase II clinical trial was performed to assess the safety and efficacy of blocking IL-4 with pascolizumab in patients with PTB receiving standard combination therapy (https://www.clinicaltrials.gov/ct2/show/NCT01638520?term=NCT01638520&cond=tuberculosis&draw=2&rank=1). The trial was scheduled to be completed in July 2017. However, the latest results have not been published (ClinicalTrials.gov identifier: NCT01638520). Recombinant β-glycan (type III TGF*β* receptor) or small interfering RNA targeting TGFβ1 was used in the mouse TB model, which blocked TGFβ, enhanced the Th1 type immune response, increased the expression of IFN-γ, IL-2, NO, and iNOS, and decreased lung bacterial counts, and downregulated IL-4 (Hernández-Pando et al., 2006). Blocking the IL-10 receptor in chronic *Mtb*-infected mice with McAb could relieve the inhibition of IL-10 on macrophage activation and Th1 immune responses, increase T cell recruitment in the lung and IFN-γ production, decrease bacterial load in the lung, and improve mouse survival rate (Beamer et al., 2008). The treatment of a mouse TB model with anti-IL-17A mAb could block massive neutrophil recruitment and tissue damage caused by Th17 overreaction (Segueni et al., 2016). Thus, maintaining the Th1-Th17 balance during TB treatment is crucial for promoting anti-TB immunity and avoiding inflammatory tissue damage. Denileukin diftitox, a recombinant fusion protein of diphtheria toxin active domain and IL-2, induced diphtheria toxin to kill CD25 cells expressing the high-affinity IL-2 receptor through the binding of IL-2 moiety, to reduce the frequency of Treg and myeloid-derived suppressor cells (MDSCs, CD11b+GR1HI) frequency, improve pathological damage, reduce the number of viable bacteria, and inhibit the spread of *Mtb* in a TB mouse model (Gupta et al., 2017). In addition, blocking PD-1 decreased the number of T cells expressing PD-1 and rescued T cells producing *Mtb*-specific IFN-γ from apoptosis and increased their survival, which may reverse T cell depletion in patients with TB (Singh et al., 2013).

## 3 Tuberculosis Therapeutic Vaccines

TB therapeutic vaccines can restore immune balance, inhibit immune damage, improve immunity, and inhibit or kill *Mtb* by regulating or selectively inducing the potential of the immune system of *Mtb*-infected people. The clinical application of vaccines is simple, convenient, economical, and has few side effects.

### 3.1 Inactivated TB Vaccines

Among the inactivated TB vaccines prepared by heat-killed non-tuberculous mycobacteria, *M. vaccae* (MV), and *M. phlei* F.U.36 (termed Utilins) have been certified for TB treatment [MV: Certificate No, (1999) S-03; F.U.36: Approval No, S20040068]. Previously, F.U.36 was mostly used for other immunocompromised diseases. In addition, *M. indicus pranii*(MIP), *M. kyogaense* sp. *Nov.* (DAR-901), RUTI, *M. smegmatis* vaccines, and MV used for latent TB infection (LTBI) have entered clinical research, and no further research on acellular *M. smegmatis* inactivated vaccines has been carried out after the completion of the phase II clinical trials in China.

MV, made from inactivated *M. vaccae*, is an immunomodulator used in the adjuvant treatment of active TB. The MV vaccine protected against pulmonary *Mtb* infection in the lungs in a mouse model (Gong et al., 2020). A meta-analysis showed that MV could effectively improve the sputum bacterium-negative rate. However, the effects on the absorption of lesions, cavity closure, and mortality are inconsistent, which may be due to the disunity of the frequency and interval of the MV application. Therefore, it is necessary to determine the most effective drug delivery scheme and the long-term effects of MV (Huang and Hsieh, 2017). A phase III, randomized, double-blind clinical trial of the treatment of LTBI with MV carried out in 2013 showed a trend of reduced TB incidence with low adverse reactions. However, the data of the completed phase III trial have not been published (ClinicalTrials.gov Identifier: NCT01979900). Another phase II trial showed that *Mtb* in sputum smears was eliminated significantly in patients with TB after one month of treatment with an MV (V7) pill, but the long-term effects still need to be observed (ClinicalTrials.gov Identifier: NCT01380119) (Efremenko et al., 2013).

The MIP (or Mw) vaccine, which is made of heat-inactivated nonpathogenic *M. indicus pranii*, can induce Toll-like receptor signaling pathways to activate innate immunity and stimulate T cell immune response (Das et al., 2016). A phase III clinical trial and meta-analysis showed that the MIP vaccine could improve the sputum-negative rate of patients with TB without adverse reactions (ClinicalTrials.gov Identifier: NCT00265226) (Sharma et al., 2017). However, MIP had no significant therapeutic effect but showed serious side effects in a phase III clinical trial of TB pericarditis (two-thirds of the subjects were TB-HIV coinfection). Abscess appeared at the injection site in 15% of patients, and the incidence of Kaposi’s sarcoma in HIV-positive patients was higher (ClinicalTrials.gov Identifier: NCT00810849) (Mayosi et al., 2014).

The DAR-901 vaccine (also known as Mk) is an immunotherapeutic agent made of heat-inactivated *M. kyogaense* sp. *nov*. Phase I and II clinical trials have shown that intradermal injection of DAR-901 is prone to form a long-term scar, and 3–12 times injection of DAR-901 combined with chemotherapy could improve sputum bacterium-negative rate, promote lesion absorption, and enhance the Th1 cytokine response (von Reyn et al., 2017; Lu and Miao, 2019). In addition, a phase II clinical trial of DAR-901 tablets showed that it could increase the sputum-negative rate and had high safety when combined with chemotherapy for one month in treating patients with TB and MDR-TB (ClinicalTrials.gov Identifier: NCT01380119) (Gong et al., 2018).

RUTI is a vaccine prepared by breaking and detoxifying *Mtb* H37Rv cultured under hypoxic conditions, low pH, and poor nutritional conditions, and then embedded in liposomes (Cardona, 2006). It can induce a humoral immune response to multiple antigens and a Th1/Th2/Th3 mixed cellular immune response with no local or systemic toxicity (Cardona, 2006). RUTI alone is ineffective in an animal model of active TB, but may also lead to immune injury. However, infected animals treated with RUTI after chemotherapy may achieve good results (Cardona et al., 2005). The results of phase I and phase II clinical trials of RUTI combined with chemotherapy in the treatment of LTBI showed that the vaccine could effectively induce a cellular immune response in LTBI volunteers. However, the adverse reactions correlated positively with the dose, and the injection site was prone to nodules (Nell et al., 2014). As an adjuvant agent of MDR-TB chemotherapy, the safety and immunogenicity of RUTI have been validated through a phase IIa clinical trial (ClinicalTrials.gov Identifier: NCT02711735).

### 3.2 TB Subunit Vaccines

TB subunit vaccines, prepared from part of the cellular components of *Mtb*, can also be used for adjuvant treatment of patients with TB and preventive therapy for LTBI. Bacillus Calmette-Guerin (BCG) polysaccharide nucleic acid injection (BCG-PSN, trade name SIQIKANG) is the only currently licensed immunotherapeutic vaccine against TB (Approval No: S20020019), but it has been mainly used in non-TB immunocompromised diseases in recent years (Nasr et al., 2018; Yan et al., 2019). The other four recombinant protein vaccines (*Mtb*72f/AS01, H56/IC31, ID93/GLA-SE, and AEC/BC02) have entered phase І or phase II clinical trials.

*Mtb*72f/AS01E is prepared with a recombinant chimeric protein (M72) of Mtb39 and Mtb32, adjuvanted with AS01E to boost the immune response (Van Der Meeren et al., 2018). Phase I/IIa clinical trials showed that *Mtb*72f/AS01E was well tolerated clinically and elicited strong M72-specific humoral immune responses and CD4^+^ T cell responses, but weak CD8^+^ T cell responses (ClinicalTrials.gov Identifier: NCT00730795 and NCT00397943, respectively) (Leroux-Roels et al., 2013). Subsequently, phase II b clinical trials showed that it could provide 54% protection for HIV-negative LTBI adults to achieve low PTB incidence (ClinicalTrials.gov Identifier: NCT01755598) (Van Der Meeren et al., 2018).

H56:IC31 is a recombinant fusion protein of three antigens (*Mtb* Ag85B, ESAT-6, and Rv2660c) formulated in adjuvant IC31 (Luabeya et al., 2015). Studies have shown that vaccination with the H56:IC31 vaccine prevented bacterial reactivation and significantly reduced bacterial load in LTBI or active TB mice or NHP models compared with the control group (Aagaard et al., 2011). The phase І/II clinical trials showed it was safe and could induce antigen-specific IgG and CD4^+^ T cell response expressing Th1-type cytokines. TNF-α+IL-2+H56-specific memory CD4^+^ T cells can be detected by low-dose vaccination in *Mtb*-infected individuals (ClinicalTrials.gov Identifier: NCT01967134) (Luabeya et al., 2015). However, the therapeutic effect of H56:IC31 requires further evaluation in clinical trials. ID93/GLA-SE is a recombinant fusion protein of four antigens (*Mtb* Rv2608, Rv3619, Rv3620, and Rv1813) formulated with glucopyranosyl lipid adjuvant in a stable emulsion (GLA-SE, a TLR-4 activator) (Baldwin et al., 2016). This vaccine combined with chemotherapy induced a strong and durable Th1-type cellular immune response, prolonged survival time, decreased bacterial burden in organs, reduced pathological damage, and enhanced the chemotherapy effect in mouse and monkey models with TB (Bertholet et al., 2010; Baldwin et al., 2021). In addition, a stable, inhalable dry powder version of ID93/GLA-SE has already been developed as an alternative to injectable administration (Gomez et al., 2021). At present, phase I clinical trials of this vaccine is underway, however, participants have not been recruited (ClinicalTrials.gov Identifier: NCT03806686 and NCT03806699, respectively).

AEC/BC02 is also a freeze-dried recombinant fusion protein of Ag85B, ESAT6, and -CFP10 combined with adjuvant BC02 based on BCG-derived cytosine-phosphate-guanine and aluminum salt (Lu et al., 2015). This new vaccine has a significant inhibitory effect on latent *Mtb* and reduces the lesions in various organs in guinea pigs, and has a preventive and protective effect on the guinea pig LTBI model (Lu et al., 2015). Phase I clinical trials that focused on the tolerance of the human body to this vaccine have been completed, but the results have not yet been published (ClinicalTrials.gov Identifier: NCT03026972). Additionally, a phase Іb clinical trial to evaluate the safety and immunogenicity of AEC/BC02 in healthy adults is underway, however, participants have not been recruited (ClinicalTrials.gov Identifier: NCT04239313).

### 3.3 DNA Vaccines

TB DNA vaccine constructed by encoding genes of *Mtb* protective antigens and a eukaryotic expression vector has become a promising strategy for developing an effective vaccine against TB. It does not only efficiently induce humoral immunity and Th1-type cell immune response, but also elicits specific cytotoxic T lymphocyte response (Liang et al., 2016; Liang et al., 2017). GX-70, consisting of four *Mtb* antigen plasmids (specific antigens not disclosed) and a recombinant Flt3-ligand, is the only TB DNA vaccine that can be used in clinical trials. Phase І clinical trials of GX-70 will be evaluated in PTB patients with at high risk for treatment failure or recurrence (ClinicalTrials.gov Identifier: NCT03159975). However, this study was withdrawn because of unconfirmed research expenses (Gong et al., 2018).

The Ag85a/b chimeric DNA vaccine, which completed the preclinical research and pilot process could induce moderate antibody levels, enhance Th1 cellular immune responses, and reduce lung tissue lesions and organ colony number *via* electric induction in a mouse TB model (Liang et al., 2012). The safety evaluation did not reveal any adverse reactions and preparation for application for clinical trial approval is underway.

Many new TB therapeutic vaccines are still in the preclinical stage. They were constructed with different *Mtb* antigens and new vaccine delivery systems, such as LT69 (HspX, ESAT6, Ag85B, and Mtb8.4 recombinant fusion protein) (Niu et al., 2015) and LT70 (Rv2626, ESAT6, Ag85B, and Mtb8.4 recombinant fusion protein) subunit vaccines (Chen et al., 2017), hsp70/CD80 chimeric DNA vaccine (Yan et al., 2013), and peptide-based ACP and MP3RT vaccines (Gong et al., 2021a; Gong et al., 2021b). These vaccines have strong immunogenicity and can significantly reduce the bacterial load in animal experiments (Shi et al., 2004; Niu et al., 2015; Liu et al., 2016). However, the immunotherapeutic effects of these vaccines against TB need to be further validated in clinical trials. Most of the TB therapeutic vaccines were combined with chemotherapy for the immunotherapy of patients with TB, and no severe adverse reactions related to the vaccines were observed. However, attention should be given to the “Koch phenomenon” problem caused by the direct application of vaccines that may induce the release of cytokines such as TNF-α when used in the preventive treatment of LTBI populations. Therefore, the immune intervention strategies of chemotherapy followed by vaccination or a combination of vaccination and chemotherapy should be further studied.

## 4 Chemical Agents

Despite the availability of antibiotics, effective treatment regimens still require long-term multidrug combinations. The discovery of new-generation antibiotics may still face problems related to drug toxicity and resistance. Current chemotherapy regimens are mainly aimed at sterilization and cannot directly reduce the host pathologic inflammatory response associated with TB. In recent years, the use of chemical agents to enhance the host immune regulatory response to *Mtb* has become an attractive approach, as it reduces the risk of drug resistance and clinical complications. Therefore, chemical agents that enhance the antibacterial activity and accelerate the decline of inflammation in the host can be considered as adjuvant therapy to exert immunotherapeutic activity to improve the clinical therapeutic effect of TB. At present, several chemical agents have reported immunotherapeutic effects against TB. Vitamin D, quercetin, and polyvinylpyrrolidone have been used in clinical studies. Some chemical drugs used in the clinical treatment of other diseases (such as the asthma drug zileuton, the anticancer drugs imatinib, everolimus, tacrolimus, and ridaforolimus; the diabetes drug metformin) are undergoing preclinical research on anti-TB.

### 4.1 Vitamin D

Vitamin D and its active metabolite 1α, 25-dihydroxy-vitamin D3 [1α, 25(OH)_2_D3], play an important role in host immune defense against *Mtb*. 1α,25(OH)_2_D3 can inhibit *Mtb* growth and secretion of pro-inflammatory cytokines in PBMCs and induce LL-37 or autophagy-related proteins Beclin-1 and Atg5 to mediate killing effects (Ramos-Espinosa et al., 2018). In addition, they have chemotactic activity on multinuclear leukocytes, activate immune cells to migrate outward, and enhance the killing effect of macrophages on *Mtb* (Agerberth et al., 2000; Martineau et al., 2007; Yuk et al., 2009). Studies have shown that vitamin D receptor activation by vitamin D administration could inhibit *Mtb*-induced bone destruction (Deng et al., 2021). Mourik *et al*. treated the mouse TB model with immunotherapy consisting of all-trans-retinoic acid, 1,25(OH)_2_-vitamin D3, and α-galactosylceramide in combination with chemotherapy, which showed lower *Mtb* loads after 5 weeks of treatment and a significantly shortened treatment course, as well as a reduction in TB recurrence (Mourik et al., 2017). However, Bekele *et al*. found that the application of vitamin D3 and phenylbutyrate combined with chemotherapy in the treatment of PTB could improve clinical symptoms and reduce complications, but had no effect on bacterial clearance in sputum (Bekele et al., 2018). The meta-analysis showed that the inconsistent clinical research results of vitamin D might be due to large differences in clinical trial design, patient characteristics, dosage, time, and study endpoints. Most research results showed that vitamin D supplementation did not improve the sputum bacterium-negative rate of patients and had good safety and tolerance, but three cases had severe adverse reactions (Wallis and Zumla, 2016). At present, the effect of vitamin D combined with chemotherapy on drug-sensitive TB is unsatisfactory, but the sputum bacterium-negative rate seems to be significantly improved in a minority of patients with MDR-TB (Wallis and Zumla, 2016). Therefore, it is necessary to further clarify its effectiveness and safety by establishing strict clinical trial protocols. Currently, several clinical trials of vitamin D as an adjunct to treat or prevent TB are ongoing, but participants are yet to be recruited (ClinicalTrials.gov Identifier: NCT01992263, NCT02880982, NCT02968927, NCT02464683).

### 4.2 Quercetin and Polyvinylpyrrolidone

Quercetin and polyvinylpyrrolidone (QP) are capillary stabilizing agents and antioxidants with immunomodulatory activities. It can promote the growth of endothelial cells, induce the activation of microcirculation in inflammation sites, and reduce inflammation and blood coagulation (Butov et al., 2016). QP combined with chemotherapy to treat patients with APTB rapidly improved the clinical symptoms and signs, significantly reduced the sputum bacteria and pulmonary cavity, increased lesion absorption, decreased expression of IL-1β and TNF-α with a substantial increase in IL-4, as well as an increase in nitric oxide (NO) at the inflammatory site with no side effects, suggesting that QP might be a new potential immunotherapy (Butov et al., 2016). Currently, 3D printed medicated skin patches containing QP have been developed, which could provide an appropriate therapeutic drug concentration and satisfactory sustained release profiles in the treatment of PTB (Chaudhari et al., 2021).

### 4.3 Bergenin

Bergenin is a natural compound extracted from fresh leaves of the genus *Bergenia*. It activates the MAPK, ERK1/2, and SAPK/JNK pathways in *Mtb*-infected macrophages, selectively induces the secretion of IFN-γ, TNF-α, IL-12, IL-17 expressed from CD4^+^ and CD8^+^ T cells, and promotes NO production for the clearance of *Mtb* in mouse TB models (Kumar et al., 2019). Bergenin combined with isoniazid in an animal model of MDR-TB could significantly reduce the pathological damage and bacterial load, and shorten the treatment time (Kumar et al., 2019). Therefore, bergenin may be a potential immunomodulator for the treatment of TB.

### 4.4 Allicin

Allicin is a major biologically active component of freshly crushed garlic, which has antibacterial activity against *Mtb* and can significantly reduce the bacterial load in the lungs of the mouse TB model (Dwivedi et al., 2019). As a potential immunomodulator against TB, allicin can also enhance the activation of the MAPK and SAPK/JNK pathways of *Mtb*-infected mouse peritoneal macrophages to produce various effectors, such as IL-1β and IL-12. In addition, allicin treatment could selectively induce Th1 responses to resist the invasion of *Mtb* and inhibit the phosphorylation of p38-MAPK to reduce the expression of TNF-α and IL-10 (Oosthuizen et al., 2017; Dwivedi et al., 2019). Although allicin can improve cellular immunity, humoral immunity, and nonspecific immunity, its efficacy in the adjuvant treatment of TB requires further study.

### 4.5 Ursolic Acid and Oleanolic Acid

Ursolic acid (UA) and its isomer oleanolic acid (OA) are representative pentacyclic triterpenoids, which have a direct inhibitory effect on mycobacteria. The immunoregulatory effect against *Mtb* of UA and OA mainly depends on the production of NO and reactive oxygen species, inhibition of TGF-β expression, induction of TNF-α expression, and activation of macrophages (López-García et al., 2015). It is well established that UA and OA can be recognized by CD36 and TGR5, respectively, to induce the overexpression of these two cell membrane receptors and activate TLR2/1, TLR2/6, or TLR4/6 heterodimer, which leads to the inhibition of nuclear factor κB (NF-κB) activation and secretion of pro-inflammatory cytokines such as TNF-α, IL-6, and IL-1β (López-García et al., 2015). The specific mechanisms of UA and OA in *Mtb-*infected macrophages should be further studied.

### 4.6 Chicoric Acid and Retinoic Acid

Chicoric acid (CA) is a phenolic compound that stimulates phagocytosis, enhances immune function, and has anti-inflammatory and anti-oxidative effects (Wu et al., 2018). Retinoic acid (RA), a natural oxidation metabolite of vitamin A, has various stereoisomers, including all-trans RA, 13-cis RA, and 9-cis RA (Gundersen et al., 1997). They exert anti-inflammatory effects through the retinoic acid receptor system. For example, the treatment of infected U937 macrophages with 13-cis RA and CA showed a significant increase in NO production, CD14, and HLA-DR expression and also prevented the intracellular survival of *Mtb* (Abd-Nikfarjam et al., 2018). Although both 13-cis RA and CA had obvious inhibitory effects on the growth of *Mtb*, the inhibitory effect of high concentrations of CA was significantly greater than that of RA (Abd-Nikfarjam et al., 2018). Therefore, the anti-TB therapeutic effects of CA and RA requires further research in animal models.

### 4.7 Curcumin

Curcumin is a polyphenolic substance that enhances *Mtb* clearance by differentiated THP-1 human monocytes and primary human alveolar macrophages. As an inducer of caspase-3-dependent apoptosis and autophagy, curcumin mediates macrophage anti-*Mtb* function in part through NF-κB activation (Bai et al., 2016). Although curcumin is safe in most cases, its anti-TB effect needs to be demonstrated in animal models.

### 4.8 Loperamide

Loperamide, a benpiperidine derivative that targets μ-opioid receptors and calcium channels, has an immunomodulatory effect and can induce autophagy, and enhances the anti-*Mtb* activity of alveolar macrophages by upregulating the expression of bactericidal/permeability-increasing protein (BPI) and antimicrobial peptide LL37 genes to reduce the bacterial load significantly (Juárez et al., 2018). Moreover, loperamide reduces the production of pro-inflammatory cytokines (IL-6, TNF-α, MCP-1, and IFN-γ) in mouse macrophages by blocking intracellular calcium influx to avoid inflammatory damage. In addition, loperamide can induce the production of TNF-α and prostaglandin E2 in endothelial cells and PBMCs by activating μ-opioid receptors (Juárez et al., 2018). Therefore, loperamide has potential immunomodulatory effects, supporting its use in the treatment of TB.

### 4.9 Phosphatidylinositol Mannosides

Phosphatidylinositol mannosides (PIMs) are a type of *Mtb* cell wall-associated lipids. It is the structural basis for the synthesis of lipomannan (LM) and lipoarabinomannan (LAM) (Brennan, 2003). PIMs exert an immunomodulatory effect by binding to macrophages, regulating the production of cytokines and active radicals, and stimulating early endoplasmic fusion by binding to Toll-like receptors, C-type lectins, and DC-SIGN ligands (Torrelles et al., 2006). It can also activate NKT cells together with CD1 molecules to produce IFN-γ, interact with α5β1 on CD4^+^ T cells to promote granulomatous formation and promote alveolar epithelial cell apoptosis (Rojas et al., 2006). Recent research has shown that tetraacylated phosphatidylinositol hexamannoside (Ac2PIM6), which is synthesized with stearic and tuberculostearic acid as the lipid components, could increase the production of IL-4 and IFN-γ in mouse serum and have comparable adjuvant activity (Patil et al., 2015). Therefore, the immunotherapeutic effect of PIMs on TB requires further verification.

### 4.10 Other Chemical Agents

Zileuton, a novel selective 5-lipoxygenase antagonist, can inhibit the production of the highly active inflammatory substance leukotriene; it has tracheal protection, tracheal dilation, and anti-inflammatory effects, and is currently being evaluated for its anti-TB effects in animal models (Mayer-Barber et al., 2014). Imatinib, a tyrosine kinase inhibitor used to treat cancers such as leukemia, could inhibit *Mtb* survival by promoting autophagy and acidification (Napier et al., 2011). Some drugs (everolimus, temsirolimus, and ridaforolimus) that inhibit graft rejection or have anticancer effects can reduce *Mtb* load by inhibiting the rapamycin (mTOR) pathway to promote cell autophagy (Singh and Subbian, 2018). Metformin, a drug used to treat diabetes, can improve and control *Mtb* infection in mice and reduce its severity by increasing mROS production in cells and mediating acidification of mycobacterium phagosomes; clinical trials of metformin against TB are currently underway (Singhal et al., 2014; Padmapriyadarsini et al., 2019). Glucocorticoids, such as dexamethasone, are effective in the treatment of disseminated TB, such as tuberculous meningitis, and studies have found that dexamethasone can significantly reduce the levels of IL-13 and IL-1RA (Clifford et al., 2015), however, more evidence is needed to determine whether glucocorticoids have potential immunomodulatory effects.

## 5 Cellular Therapy

Cellular therapy, as a potential adjunct therapeutic option for TB, refers to killing *Mtb* and infected cells by activating and expanding autologous or allogeneic immune effector cells *in vitro* and then transfusing them to the patient to correct immune imbalances and improve immune function (Rao et al., 2019). Animal experiments and clinical trials have shown that cellular therapy provides a promising new immunotherapy approach for the individualized and comprehensive treatment of MDR-TB, extensively drug-resistant TB (XDR-TB), and widely disseminated TB.

### 5.1 Mesenchymal Stem Cells

Mesenchymal stem cells (MSCs) are multipotent differentiation stem cells that are usually derived from the bone marrow, cord blood, and placenta (Wang et al., 2013). The advantages of their anti-inflammatory, immune regulation, tissue regeneration and repair promotion, and low immunogenicity make them promising immunotherapies for immune system disorders and immune tissue damage caused by chronic *Mtb* infection (Parida et al., 2015). The clinical trial results of autologous bone marrow-derived MSC-assisted treatment in patients with MDR-TB and XDR-TB with no response to previous chemotherapy showed that the functional immune response of the host recovered, the sputum turned negative, the lung cavity narrowed or closed, and the cure rate significantly increased, with only a few adverse reactions occurring in a few patients (Erokhin et al., 2008; Skrahina et al., 2012; Skrahin et al., 2014). Nonetheless, it has also been found that MSCs may activate dormant *Mtb* and assist *Mtb* immune escape and long-term latency in the host, thus leaving the possibility of TB recurrence (Hoagland et al., 2016). Moreover, it may induce a Th2 immune response to produce IL-4, inhibit the production of IFN-γ, form an immunosuppressive microenvironment in infected organs, and increase the susceptibility to pathogenic microorganisms, causing extrapulmonary TB (Hoagland et al., 2016; Khan A. et al., 2016). In addition, MSCs are stromal cells, rather than typical immune cell types. They usually regulate immunity and promote regeneration by secreting cytokines or growth factors, which makes the therapeutic mechanism difficult to clarify. In recent years, no new clinical data of MSCs in the treatment of MDR-TB or XDR-TB have been reported, so more studies are needed to clarify the therapeutic effect on TB.

### 5.2 γδ T Cells

γδ T cells can be divided into two subgroups depending on the different expressions of the γ and δ chains. As a major human peripheral γδ T-cell subset, Vγ9Vδ2 T cells mainly participate in the immune response by lysing cells, producing cytokines, and regulating immune cells. It has the advantages of non-major histocompatibility complex (MHC) restriction (allogeneic use), easy amplification *in vitro*, and low cost. Thus, Vγ9Vδ2 T cells have become adoptive cells for immunotherapy (Vantourout and Hayday, 2013). Several studies in primate *Mtb* infection models have shown that the PBMCs were induced by phosphate antigen combined with IL-2 injection to expand Vγ9Vδ2 T cells. Expanded Vγ9Vδ2 T cells were transplanted and then accumulated in the lungs, which could significantly reduce the bacterial load in the lungs and extrapulmonary organs, prevent the spread of *Mtb*, significantly reduce lung tissue injury, and increase the weight of animals. Amplified Vγ9Vδ2 T cell transplantation inhibits or kills intracellular *Mtb* by producing IFN-γ, perforin, and granulysin (Shen et al., 2019). In addition, expanded Vγ9Vδ2 T effector cells and IL-12 enhanced the response of CD4^+^/CD8^+^ T cells in the lung, thereby enhancing the resistance of the body to *Mtb* infection (Chen et al., 2013). At present, clinical trials of Vγ9Vδ2 T cells in the treatment of MDR-TB are underway in China but without relevant reports.

### 5.3 Cytokine-Induced Killer

Cytokine-induced killer (CIK) cells are nonrestrictive killer cells derived from human PBMCs stimulated by multiple cytokines and are amplified *in vitro* (Introna, 2017). One case of disseminated pulmonary TB was treated with chemotherapy combined with CIK immunotherapy in China, which showed sputum culture- and smear-negative results without liver damage after one month of treatment (Xu et al., 2015). Additional clinical trial data are needed to prove its effectiveness.

### 5.4 Invariant NKT Cells

Invariant NKT (iNKT) cells, a conservative subset of T cells, are restricted by CD1d and are dominated by CD4^-^CD8^-^ NKT cells (Nakamura et al., 2003). *Mtb*-infected macrophages can activate iNKT cells to release a large amount of IFN-γ, further activate macrophages to secrete TNF-α and NO, and enhance the killing effect of phagosomes and lysosomes on *Mtb* (Sada-Ovalle et al., 2008). *Mtb* infection promotes the secretion of GM-CSF by iNKT cells in a CD1d-dependent manner to inhibit *Mtb* proliferation (Rothchild et al., 2014). Transplantation of iNKT cells can also limit *Mtb* proliferation *in vivo* and may become a new cell therapy to treat TB.

In addition, macrophages produced by BCG-induced differentiation of mouse hematopoietic stem cells (HSCs) provide significantly better protection against virulent *Mtb* infection than naïve macrophages. Adoptive transplantation of *Mtb-*infected macrophages from the bone marrow of animals vaccinated with BCG to another animal also showed long-lasting anti-TB activity *in vivo* (Kaufmann et al., 2018).

## 6 Future Challenges and Prospects

As the key to controlling *Mtb* infection, the immunity of the host has made some breakthroughs in TB immunotherapy. The application of immune interventions, including immunologically active substances, therapeutic vaccines, chemical agents, and cell therapies combined with anti-TB drugs have shown satisfactory results in animal experiments or clinical trials; a few of them have been used in the clinic. However, the overall clinical treatment of TB has not changed significantly, and the goal of “ultra-short course chemotherapy” has not been achieved. The reasons may include the following four aspects: (1) The immune regulation mechanism of TB has not yet been fully elucidated. It is well known that the immune response of TB is a double-edged sword for TB treatment. Effective use of immune methods to conduct immune regulation and immune intervention to promote its physiological response and suppress its pathological effect, to seek benefits and avoid harm, is the direction for further research in the future. (2) Only a few immunotherapy preparations can be used for clinical treatment. Many new immunotherapies are still in the preclinical research stage, suggesting that there are still many hurdles to overcome in fundamental scientific discoveries to clinical application. (3) There is a lack of immunological markers that systematically reflect the biological stages of *Mtb in vivo*. It is necessary to develop new immunodiagnostic methods to help clinicians clearly understand the progress of TB, judge the therapeutic effects, and predict clinical outcomes, to provide experimental evidence for the application of immunotherapy. (4) The clinical application and research are not in-depth, and the application of immune preparations is not standardized. There is also a lack of in-depth studies on the selection, dosage, application timing, treatment course of immune agents, immune status of the hosts, and impact on immunity. Moreover, a standardized, safe, and effective combined therapy scheme has not yet been developed. The development of molecular biology and immunology will reveal the immune response mechanism of TB at the molecular and cellular levels. Given the different immune defects or immune abnormalities in patients, new immunotherapeutic agents and methods will be developed, and appropriate immunotherapeutic agents and methods will be reasonably selected for combined application with anti-TB chemotherapy to treat TB effectively. Thus, immunotherapy is expected to become another critical and promising breakthrough in controlling TB, especially MDR-TB, following chemotherapy.

## Author Contributions

JM: wrote and revised the manuscript. YL, JQL, WPG, SYW, JXZ and ZML: consulted the literatures and contributed with writing. XQW: supervised the manuscript. All authors contributed to the article and approved the submitted version.

## Funding

This work was supported by the grant from the Serious Infectious Diseases Special Foundation of China (2018ZX10731301-005) and Key Projects of Medical Innovation Engineering (18CXZ028).

## Conflict of Interest

The authors declare that the research was conducted in the absence of any commercial or financial relationships that could be construed as a potential conflict of interest.

## Publisher’s Note

All claims expressed in this article are solely those of the authors and do not necessarily represent those of their affiliated organizations, or those of the publisher, the editors and the reviewers. Any product that may be evaluated in this article, or claim that may be made by its manufacturer, is not guaranteed or endorsed by the publisher.
